# Multiplex detection of miRNAs based on aggregation-induced emission luminogen encoded microspheres†

**DOI:** 10.1039/c9ra07680h

**Published:** 2019-12-04

**Authors:** Dan Zou, Weijie Wu, Jingpu Zhang, Qiang Ma, Sisi Fan, Jin Cheng, Dan Li, Jiaqi Niu, Xiaoqing Qian, Wanwan Li, Daxiang Cui

**Affiliations:** Institute of Nano Biomedicine and Engineering, Shanghai Engineering Research Center for Intelligent Instrument for Diagnosis and Therapy, Department of Instrument Science & Engineering, School of Electronic Information and Electrical Engineering, Shanghai Jiao Tong University 800 Dongchuan Road Shanghai 200240 China dxcui@sjtu.edu.cn; National Center for Translational Medicine, Collaborative Innovational Center for System Biology, Shanghai Jiao Tong University Shanghai 200240 P. R. China; State Key Lab of Metal Matrix Composites, School of Materials Science and Engineering, Shanghai Jiao Tong University 800 Dongchuan Road Shanghai 200240 P. R. China wwli@sjtu.edu.cn; Scientific Research Center, Shanghai Public Health Clinical Center, Fudan University 2901 Caolang Road Shanghai 201508 P. R. China; Tumor Department of Xintai People Hospital 1329 Xinfu Road Xintai Shandong Province China

## Abstract

Herein, we report a multiplex detection platform based on a suspension array with aggregation-induced emission luminogen (AIEgen) barcodes for simultaneous quantitative measurement of let-7b-5p, miR-16-5p and miR-19b-3p, which are associated with gastric cancer. A detection strategy by using a flow cytometer is proposed, which utilizes AIEgen-encoded microspheres to quantify the target miRNAs, and phycoerythrin as a fluorescence reporter on the detection probes to provide quantitative signals. This multiplex assay shows good specificity for recognizing single base mismatch, and possesses excellent sensitivity with limits of detection (LODs) ranging from 0.43 to 0.76 nM for the three miRNAs. The approach could be extended to the simultaneous detection of more target miRNAs by designing specific detection probes and increasing the number of fluorescence barcodes. We could foresee it holding great potential in future laboratory research and clinical applications due to its flexibility, strong multiplexed ability and good detection performance.

## Abstract 2

A miRNA multiplex detection assay based on aggregation-induced emission luminogen encoded microspheres.

## Graphical abstract

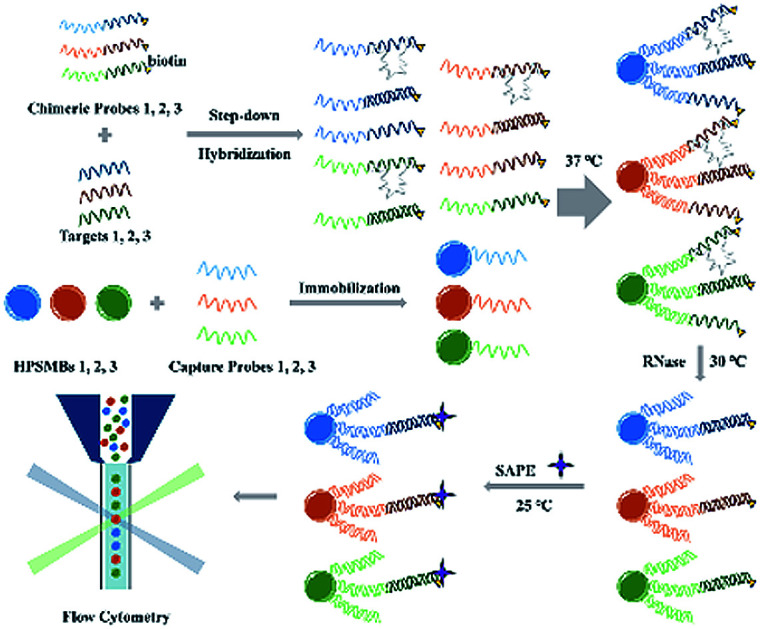

## Introduction

MicroRNAs (miRNAs) are small non-coding RNA molecules (18–25 nucleotides) that regulate gene expression by the degradation of target mRNAs or the inhibition of protein translation.^1,2^ To date, plenty of miRNA biomarkers have been screened for diagnosis and prognosis of cancers.^3–13^ However, the inherent characteristics of miRNAs, such as instability, high similarities among family members and low abundance, increase the difficulties of miRNA single-plex detection accuracy. Therefore, miRNA multiplex detection is beneficial to overcome the clinical sensitivity and specificity limitations. Although many miRNA detection technologies have been developed including northern blotting,^14^ microarrays,^15^ reverse-transcription polymerase chain reaction (RT-PCR),^2,16,17^ Isothermal amplification technology^18,19^ and next-generation sequencing technology,^20^ these methods have some drawbacks, such as requiring target amplification, being poorly flexible and reproducible, and not being easily multiplexed.^21^ In contrast, suspension array technology has been widely applied in multiplex detection with excellent flexibility and strong multiplex ability.^22,23^

Suspension array technology commonly uses optically encoded microspheres as solid-supports and tracking codes to allow for the parallel detection of multiple targets. Traditional organic dyes and quantum dots (QDs) are the most popular fluorophores for constructing optically encoded microbead libraries because of their large encoding capacity and high-speed decoding of barcodes. However, both traditional organic dyes and QDs always exist aggregation-induced quenching phenomenon (ACQ) effect under high concentration during the preparation of encoded microbeads.^24,25^ A new kind of fluorescent material AIEgens has been reported in recent years, and has attracted considerable interests in constructing various novel chemosensors and biosensors due to their excellent optical properties, such as high brightness, excellent photobleaching resistance, low background interference and great biocompatibility except for unique AIE effect comparing with traditional organic dyes and QDs.^26,27^ Hence, AIEgens also will be ideal candidate for constructing optical encoded microspheres to establish miRNA multiplex detection platform.

Herein, AIEgens-encoded microspheres were firstly prepared by incorporating an AIEgen (hexaphenylsilole, HPS) into polystyrene-*co*-maleic anhydride (PSMA) microspheres through facile Shirasu porous glass (SPG) membrane emulsification method.^28,29^ Based on the as-prepared AIEgens-encoded microspheres, a novel suspension array platform was established to achieve multiple detections of let-7b-5p, miR-16-5p and miR-19b-3p, which are associated with gastric cancer.^13^ Capture probes and chimeric probes for these three miRNAs were designed based on the general method proposed in this paper. To achieve higher detection sensitivity, several parameters were studied. The results of specificity tests showed that these multiplex assays could achieve excellent specificity even to recognize single base mismatch. Eventually, triplex detection assay was performed and showed high sensitivity with LOD ranging from 0.43 to 0.76 nM for the three miRNAs.

## Experimental section

### Apparatus

The hybridization reaction between target miRNAs and chimeric probes was performed in a thermal cycler (LongGene, MyGene™ L, China). Oscillator (VELP, ZX4, Italy) and shaker (Thermo Electron Corp, Forma Orbital Shaker, America) were needed in the hybridization reaction between capture probes and chimeric probe-miRNAs at 37 °C as well as the RNase digestion at 30 °C. Filtration device (Millipore, America) was used in the washing operations. Flow cytometer (Beckman, CytoFLEX, USA) was used for the barcoding and decoding of AIEgens-encoded microspheres, and analysis of samples after finishing hybridization.

### Materials and reagents

All chemicals were used as received, without further purification. Both capture probes and target miRNAs were synthesized by Sangon Biotech (Shanghai, China), and chimeric probes were synthesized by GENEWIZ (Suzhou, China). Specifically, the capture probe was modified NH_2_C_6_ at the 5′ end and was purified by HPLC. Both capture probes and target miRNAs were dissolved in nuclease-free water. The chimeric probe included a DNA fragment and an RNA fragment from 5′ to 3′ end, and the 5′ end was biotinylated. Chimeric probes were dissolved in 1× Tris–EDTA (TE) buffer which was purchased from Sangon Biotech (Shanghai, China). Nuclease-free water, 0.5 M ethylenediaminetetraacetic acid (EDTA, pH 8.0), 3 M sodium acetate trihydrate (NaAc, pH 5.2), 1 M Tris(hydroxymethyl) aminomethane (Tris–HCl, pH 7.4), Tween-20 and 10% sodium dodecyl sulfate (SDS) were purchased from Beyotime (Shanghai, China). 0.5 M EDTA (pH 8.0), 3 M NaAc (pH 5.2), 1 M Tris–HCl (pH 7.4) and Tween-20 were used to prepare the hybridization/wash buffer, which is a pH 7.7 buffer consisting of 10 mM Tris, 200 mM sodium acetate, 5 mM EDTA, and 0.05% Tween 20. Diethyl 2,5-di(thiophen-2-yl) terephthalate (MES, 0.1 M, pH 4.7) was purchased from Yuanye Bio-Technology (Shanghai, China). 1-Ethyl-3-(3-dimethylaminopropyl) carbodiimide (EDC) was purchased from SIGMA. Streptavidin and R-Phycoerythrin Conjugate (SAPE, 1 mg mL^−1^) was purchased from Thermo Fisher. RNase ONE™ ribonuclease (M4265, 10 U μL^−1^) was purchased from Promega.

### Sample preparation

#### Preparation of AIEgens-encoded microspheres

AIEgens-encoded microspheres were prepared through SPG membrane emulsification method using SPG membrane with the pore size of 5 μm.^22^ Firstly, different amounts of HPS and 0.5 g of PSMA were dissolved in 8 mL of toluene to obtain dispersed phase. The amounts of HPS in dispersed phase for three barcodes are 0.05 mg, 0.25 mg and 1.25 mg, respectively. In the meantime, a continuous phase was prepared by dissolving 1 g of SDS into 200 mL of water. Then uniform oil-in-water droplets were continuously formed on the surface of SPG membrane by pressing dispersed phase into continuous phase under proper nitrogen pressure at room temperature. After toluene in oil-in-water emulsion was evaporated for 24 h, the solidified HPS-encoded microspheres (HPSMBs) were purified by centrifugation with water and ethanol separately three times to remove excess SDS. Finally, HPSMBs were resuspended in poly(styrene-*co*-maleicanhydride) (PBST) buffer for later use.

#### Immobilization of the capture probes onto microspheres

To perform suspension assays for target miRNAs, capture probes were immobilized onto the carboxyl modified surface of the microspheres with chosen fluorescent barcodes. Before the coupling reaction, capture probes were firstly denatured at 95 °C for 10 min to remove the secondary structures and dimers, ensuring that capture probes could maintain the single-stranded structure before the hybridization reaction. Selected encoded microspheres were firstly washed and carboxyl activated. Specifically, microspheres storage solution (included one million microspheres) and 400 μL PBST buffer were mixed and centrifugated at 14 000 × *g* for 4 min, and then the supernatants were removed. Such operations were repeated three times. After washing steps, 400 μL activation buffer MES was added to the dry microspheres followed by the centrifugation at 16 000 × *g* for 4 min. Appropriate amount of denatured capture probes and 10 μL of freshly prepared EDC solution (200 mg mL^−1^ in MES buffer) were quickly mixed with the acquired dry microspheres in 50 μL MES buffer. After 30 min incubation on a shaker at 600 rpm in the dark, the other 5 μL of freshly prepared EDC solution (10 mg mL^−1^ in MES buffer) was quickly added to the above mixture followed by a 30 min incubation on a shaker at 600 rpm in the dark. After that, excess EDC and capture probes were removed by the subsequent washing operations. 400 μL PBST buffer was added into the above regents and centrifugated at 14 000 × *g* for 4 min. Such washing steps were repeated twice followed by another wash by adding 400 μL 0.1% SDS to the prepared microspheres (14 000 × *g*, 4 min). Finally, the microspheres attached with capture probes were resuspended in a proper volume of 1× TE buffer and stored at 4 °C until further use.^22^

#### Suspension single assay of target miRNAs using flow cytometer

Firstly, target miRNA solutions in different concentrations (0, 0.001, 0.01, 0.1, 1, 10, 100 nM) were prepared with nuclease-free water, and chimeric probe solution was resuspended at 1 μM with 1× TE buffer firstly and diluted to some proper concentrations with hybridization buffer. Then the hybridization reaction between target miRNAs and chimeric probes was performed in mixtures containing 16.3 μL hybridization buffer, 2.5 μL miRNA solution in different concentrations and 1.25 μL chimeric probe solution with specific concentration. After quick vortex and spin, the hybridization samples were put in a thermal cycler. In brief, the hybridization was firstly performed at 90 °C for 3 min, and then incubated from 80 °C to 60 °C with the dropping rate at −1 °C per 6 min. When the temperature jumped to 37 °C, 4 μL microspheres solution coupled with capture probes was quickly added to the hybridization sample and incubated for 30 min at 37 °C on a shaker at 600 rpm. Then 2.5 μL RNase solution with specific concentration was added to the reaction sample. After 30 min of RNase digestion at 30 °C on a shaker at 600 rpm, samples were transferred to a PCR plate and washed three times with wash buffer to remove the excess probes, enzymatic residue and RNase. Then 100 μL SAPE solution with specific concentration was added to each well for staining, and the mixture was incubated at 25 °C on a shaker for 1 min at 1000 rpm and 29 min at 500 rpm. After that, each well was washed three times with wash buffer to remove the excessive SAPE. Finally, microspheres with probes hybridized on their surfaces were resuspended in 100 μL wash buffer and transferred to flow cytometer tubes for decoding. For each assay, about 1000 size-gated microbeads were read for analyzing and decoding by mapping the fluorescence profiles from 525/40 BP and 660/20 BP detection channels of the flow cytometry. The median fluorescence intensity value (MFI) of SAPE (PL peak at ∼580 nm) was positively correlated to the concentration of the corresponding target miRNA and was detected by the 585/42 BP detection channel.^22,23,30^

#### Specificity evaluation

The specificity experiments were performed according to the following steps. Adding equal amounts of single capture probe (capture 1, 2 or 3, see Table 1) (100 pmol/10^6^ microspheres) and single chimeric probe (chimeric 1, 2 or 3, see Table 1) (0.01 μM) to the reaction samples as described above, and then adding equal amounts of DEPC water, target 1, 2, 3, 4, 5 (see Table 1) (5 pM) to the above reaction samples respectively, the steps were conducted the same as “suspension single assay of target miRNAs using flow cytometer”.^21^

**Table 1:** Sequences of oligonucleotides used in this work

Name	Mark	Sequence (5′ to 3′)
hsa-let-7b-5p	Target 1	UGAGGUAGUAGGUUGUGUGGUU
	Capture 1	NH_2_–ATAAGAGAATGAAGAAGTATGA
	Chimeric 1	Biotin–AACCACACAACCUACUACCUCATCATACTTCTTCATTCTCTTAT
hsa-miR-16-5p	Target 2	UAGCAGCACGUAAAUAUUGGCG
	Capture 2	NH_2_–TGATATGTTATTGTTGAGTGAT
	Chimeric 2	Biotin-CGCCAAUAUUUACGUGCUGCUAATCACTCAACAATAACATATCA
hsa-miR-19b-3p	Target 3	UGUGCAAAUCCAUGCAAAACUGA
	Capture 3	NH_2_–AGATGATGAGATTAGATTGATTG
	Chimeric 3	Biotin–UCAGUUUUGCAUGGAUUUGCACACAATCAATCTAATCTCATCATCT
hsa-let-7a-5p	Target 4 (control 1)	UGAGGUAGUAGGUUGUAUAGUU
hsa-miR-23a-3p	Target 5 (control 2)	AUCACAUUGCCAGGGAUUUCC

#### Suspension multiplex assay of target miRNAs using flow cytometer

Multiplex assay of target miRNAs was conducted the same as the procedures in the single assay but with some adjustments. Firstly, in the hybridization sample, 2.5 μL miRNA solution contained three different miRNAs instead of single miRNA. However, the concentrations of each miRNA were the same as those in the single assay. Similarly, 1.25 μL chimeric probe solution contained three different chimeric probes in the specific concentration of each chimeric probe as described in the single assay. 4 μL beads solution contained three different encoded microspheres coupled with specific capture probes in the same number of each microsphere as that in the single assay. Secondly, because the numbers of probes and microspheres in the multiplex assay were three times those of a single assay, the amounts of RNase and SAPE were correspondingly adjusted to three times of those in the single assay.

## Results and discussion

### Design of the detection strategy

**Scheme 1:**
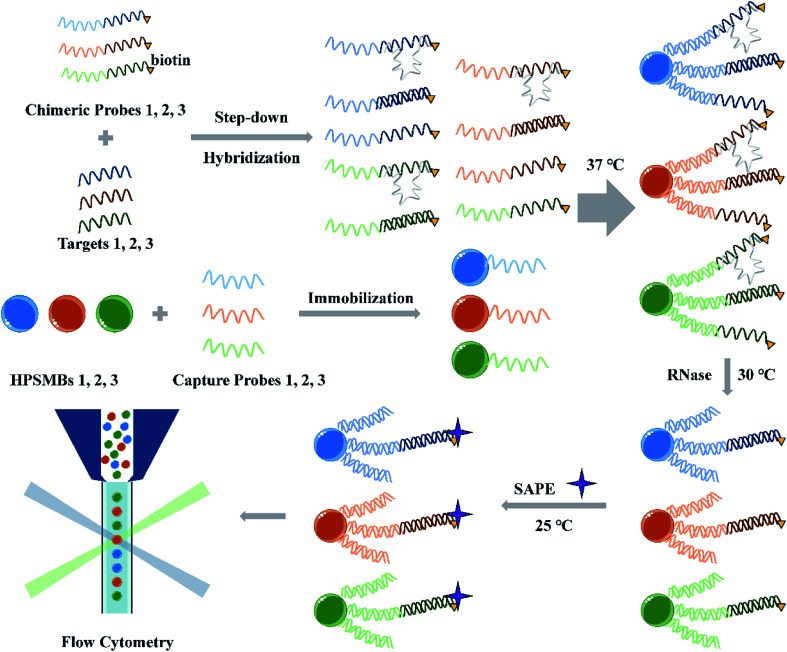
Schematic illustration of miRNA multiplex detection assay based on AIEgens-encoded microspheres.

The miRNA multiplex assay based on AIEgens-encoded microspheres is illustrated in Scheme 1. Briefly, capture probes are DNA oligomers decorated with NH_2_C_6_ at their 5′-ends and coupled onto the surface of carboxylated AIEgens-encoded microspheres by amide bond. Biotinylated chimeric probes consist of a DNA single-strand fragment complementary to the capture probe and an RNA fragment complementary to the target miRNA, respectively. In the presence of target miRNAs, the miRNAs and their corresponding chimeric probes are hybridized in a step-down thermo reaction. Then barcoded microspheres coupled with specific capture probes are hybridized with miRNA–chimeric probe complexes. RNase digestion is performed to remove any single-strand RNA which include the RNA fragments in the excess chimeric probes and mismatched single-strand RNA in our detection system, and thus ensure that only perfectly-matched structures formed by the capture probes, chimeric probes and the targeted miRNAs are protected, instead, the other acquired hybrids would be shortened to DNA duplex without RNA fragments attached by fluorophore (Scheme 1). Finally, after fluorescent reporter SAPE reacted with biotinylated chimeric probes, different fluorescent encoded microspheres with perfectly-matched probe structures were decoded and analyzed by flow cytometer. Notably, different target miRNAs can be sorted by the microspheres according to their fluorescent codes, and simultaneously the intensity of one specific target miRNA can be counted according to the signal from emitters on chimeric probes through the MFI quantifying. In contrast, in the absence of the target miRNAs, binding between chimeric and capture probes would occur, but the RNA fragment in the former probe could then be cleared by RNase digestions, leaving only the DNA hybrids without fluorescence signals. There are two key elements to the success of the assay: (1) the design of capture probes and chimeric probes. In multiplex assay, the cross reaction between different probes and different target miRNAs should be avoided to ensure a good specificity and accuracy of the multiplex assay. (2) The preparation of optically encoded microspheres. The choice of coding method and material needs to ensure high throughput of codes and low background signals, which directly affect the assay's sensitivity.

### Design of capture probes and chimeric probes

Let-7b-5p, miR-16-5p and miR-19b-3p were selected to demonstrate our miRNA multiplex assay model. The above miRNAs as plasma biomarkers are correlated with the development of human gastric cancer (proposed in our team's previous work).^13^ To detect the above miRNAs in one multiplex assay, three sets of capture probes and chimeric probes need to be designed individually. Given that the chimeric probe is partially complementary to capture probe and target miRNA respectively, once the target miRNA and the capture probe are determined, the chimeric probe is accordingly determined. Thereby, the key is to successfully design capture probes. Some principles should be followed for the design of capture probes. Firstly, the capture probes and their complemental sequences have no similarity with the target miRNAs to reduce the non-specificity hybridization. Secondly, there is no similarity among different capture probes to avoid unexpected hybridization. Thirdly, in the multiplex assay, the hybridization reactions of different probes were performed at the same temperature, and thus different capture probes should own similar melting temperature (*T*_m_) to ensure different miRNAs achieve better hybridization at the same time in the multiple assay.

Based on the above principles, the specific design steps for three capture probes matched to the three miRNAs are as follows. Firstly, preliminary screening of capture probes was conducted. Although 150 kinds of ‘anti-TAGs on beads’ are provided in the miRNA-chimeric-probe design-tool provided by Luminex, screening out sequences from 150 sequences that satisfy the above principles means too much complicated work. A simple method was tried in the presented study. Firstly, the 150 ‘anti-TAGs’ were arranged randomly to one single long sequence and then the Primer Search function of the software Primer Premier 6 was used to initially screen out the capture probes based on the following conditions: *T*_m_ was set to 35 to 45 °C and the length was set to 18 to 24 bp. Finally, 10 capture probes that were not easy to form either secondary structures or hairpin structures were screened out. After the preliminary screening, further screening of capture probes was performed. The Homology Analysis function in the software Oligo 7 was used to analyze the similarity among the 10 capture probes and the similarity between the 10 capture probes and the three target miRNAs. Eventually, three capture probes were selected by eliminating those which have more similarities with other probes and miRNAs (Table 1). Taken together, we can see that the method developed here to screen capture probes for multiplex assay owns the following two advantages. On the one hand, it exhibits high simplicity. We design the capture probes based on 150 ‘anti-TAGs’ provided by Luminex, which in fact utilizes the good characteristics of 150 ‘anti-TAGs’, so we can easily get capture probes that don't tend to form the secondary structures and hairpin structures. On the other hand, the method here is not limited to the three miRNAs mentioned in this study. Instead, it can be utilized for a broader use for other small RNAs based on the proposed assay. Moreover, selected conditions including *T*_m_ and length can be adjusted according to the needs. However, a better and more reasonable method to arrange the 150 ‘anti-TAGs’ and an optimized analysis logic for the screening steps are also expected to satisfy more utilities.

### Preparation and characterization of AIEgens-encoded microspheres

**Fig. 1:**
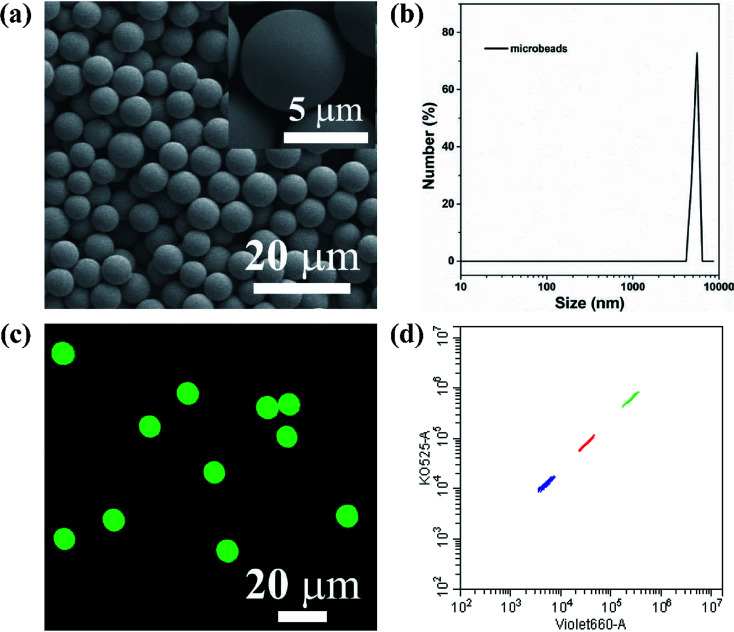
Morphology characterization and barcodes signal of HPSMBs. (a) SEM images of HPSMBs 2. (b) Dynamic light scattering of HPSMBs 2. (c) Confocal laser scanning microscopy (CLSM) luminescence images of HPSMBs 2. (d) Fluorescent barcodes signal of HPSMBs at 3 different concentration levels of HPS.

The key technology of suspension array is optically encoded microspheres with unique fluorescent barcodes signal to address various analytes for individual quantification. Therefore, sufficient coding method and coding material are needed to fulfill high-density and specific multiplexing. Herein, AIEgens-encoded microspheres were produced by inducing HPS into PSMA microspheres through SPG membrane emulsification technique using SPG membrane with a pore size of 5 μm. As shown in Fig. 1a and b, highly uniform 6 μm HPSMBs 2 with smooth surfaces and good monodispersity were successfully prepared, and the coefficient of variation (CV) is about 8% according to the statistics of 200 HPSMBs 2 in SEM images. The confocal laser scanning microscopy (CLSM) luminescence images of HPSMBs 2 present bright fluorescent intensity and good distribution of HPS inside the microspheres, indicating that HPSMBs 2 have great potential as barcodes in suspension array (see Fig. 1c). After successfully prepared HPSMBs 2 with good uniformity and unique optical properties, we generated HPSMBs according to the most popular encoding strategy of combining color and intensity, in order to obtain different optical barcodes for multiplex detection of miRNAs. We facilely synthesized three distinguishable HPS barcodes by incorporating different amounts of HPS, which were measured by flow cytometer (see Fig. 1d). The characterization results of the other two kinds of HPSMBs were shown in Fig. I and II (ESI†). Compared with QDs, there are two advantages for AIEgens as fluorophores to prepare barcodes in the present study. Firstly, the amount of HPS in barcodes is almost one-tenth of green QDs to reach the same fluorescence intensity. Secondly, the cluster of HPS barcodes is smaller and centralized compared with QDs-encoded microspheres. Thereby, the barcode cluster of as-prepared HPS barcodes are small, centralized and easily distinguishable, which could be an ideal carrier and make it possible for further multiplexed detection of miRNAs.

### Optimization of reaction parameters

**Fig. 2:**
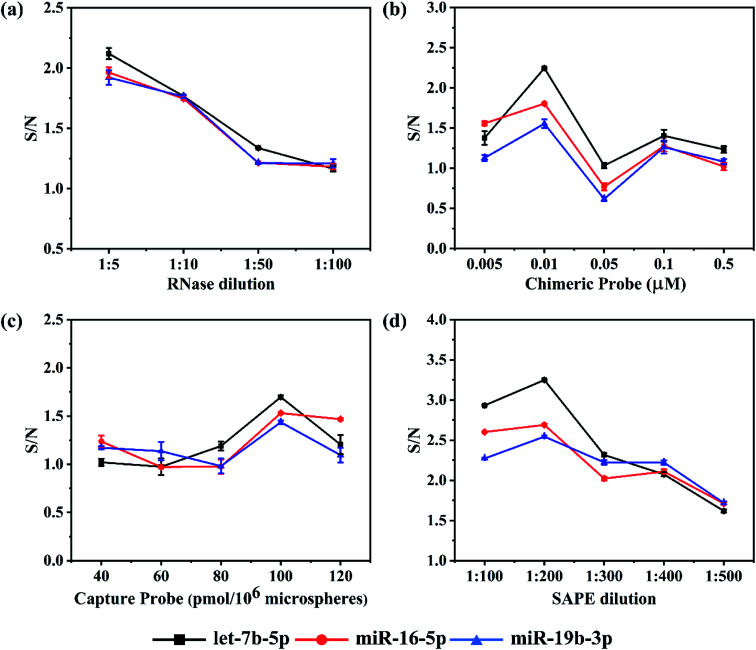
Effects of RNase, chimeric probe, capture probe and SAPE on the S/N ratio. Experimental conditions: (a) chimeric probe concentration 0.01 μM, capture probe coupling concentration 80 pmol/10^6^ microspheres, SAPE dilution 1 : 200 and microspheres density 10^4^ microspheres per well; (b) RNase dilution 1 : 10, capture probe coupling concentration 80 pmol/10^6^ microspheres, SAPE dilution 1 : 200 and microspheres density 10^4^ microspheres per well; (c) RNase dilution 1 : 10, chimeric probe concentration 0.01 μM, SAPE dilution 1 : 200 and microspheres density 10^4^ microspheres per well; (d) RNase dilution 1 : 10, chimeric probe concentration 0.01 μM, capture probe coupling concentration 100 pmol/10^6^ microspheres and microspheres density 10^4^ microspheres per well.

To achieve higher detection sensitivity, several parameters were studied to obtain optimal conditions for detecting the target miRNAs, including the dilution ratio of RNase and SAPE, the dosage of capture probes and chimeric probes (see Fig. 2). For each miRNA assay, the reaction parameters were individually optimized to ensure all of them to achieve the best hybridization results. Assay sensitivity was evaluated by comparing the S/N (Signal/Noise) ratio of 1 nM *versus* buffer control.^21^ In this assay, RNase digestion directly affects the accuracy of the detection results. In order to find a proper quantity of enzyme that ensured the sufficient RNase digestion, the RNase stock solution (10 U μL^−1^) was diluted according to the ratios of 1 : 5, 1 : 10, 1 : 50 and 1 : 100 for miRNA detection. As expected, in all three assays, both the background noise and target signal went up as the dilution folds of RNase increased. However, it should be noted that the S/N slightly decreased as the dilution ratio increased in all three assays (Fig. 2a). Moreover, there were no significant differences among the ratios for each assay. In the end, instead of 1 : 5 the ratio of 1 : 10 was chosen because there was no obvious advantage comparing the S/N of 1 : 5 with that of 1 : 10, but the cost of enzyme usage would be reduced by half.

Once the RNase dilution ratio was determined, the dosage of chimeric probes was next to be optimized. Five concentrations ranging from 0.005, 0.01, 0.05 and 0.1 to 0.5 μM were studied. We found an interesting phenomenon that the background noise in all three assays increased firstly and then decreased with an increase in concentration of chimeric probes, reaching a peak at 0.05 μM. However, there was a slight difference in the target signal among the three assays. Like the background noise, the target signal increased firstly and then decreased as the concentration of chimeric probes increased, but the chimeric 2 and 3 reached a peak at 0.01 μM, while the chimeric 1 at 0.05 μM. For all three assays, the S/N ratio reached the maximum at 0.01 μM and minimum at 0.05 μM (Fig. 2b). When the concentrations of chimeric probes are at 0.1 and 0.5 μM, both the background noise and target signal were too low to address the accurate detection results. Besides, the repeatability of the experimental results was poor. The reason for such phenomenon may be that once the chimeric probes in the hybridization reaction are largely excessive, they are so easier to form secondary structures or perform cross hybridization that the target miRNAs could not be captured completely. In addition, the mismatched structures would be cleared in the RNase digestion reaction, finally resulting in weaker fluorescent signals at higher concentrations of chimeric probes compared with relatively lower concentrations. Therefore, it is important to choose an appropriate concentration of chimeric probe. As a result, 0.01 μM was determined as the optimal concentration for chimeric probes.

In this detection platform, the fluorescent signal read by the flow cytometer is the average of the fluorescent signals for all the encoded microspheres. In theory, the more capture probes coupled on the encoded microspheres, the higher the fluorescent signal will be. In order to investigate the actual effect of the number of capture probes on the microspheres, five values including 40, 60, 80, 100 and 120 pmol/10^6^ microspheres were set. As expected, in the lower concentrations, both the background noise and target signal in the three assays were strengthened as the number of capture probes increased. However, in the higher concentrations, both the background noise and target signal tended to reach the plateau or even reduced. For all the three assays, the S/N ratios increased as the number of capture probes increased from 40 to 100 pmol/10^6^ microspheres, but began to reduce once over 100 pmol/10^6^ microspheres (Fig. 2c). This phenomenon could probably be attributed to the fact that once the capture probes are largely excessive, the more mismatch hybridization will happen among the capture probes. In addition, the aggregation-induced fluorescence quenching effect may occur if the fluorescent modules are too dense on the surface of microspheres. Eventually, 100 pmol/10^6^ microspheres were determined for all three assays.

Based on the fact that the fluorescent signal from emitter-conjugated chimeric probes is utilized to quantify the concentration of target miRNA, it is important to use proper amount of SAPE fluorescent dye. If the amount of SAPE is too excessive, the background noise may become too large, while if insufficient dye is used, not enough signals could be generated to mark the perfect hybridization structures. Five dilution ratios including 1 : 100, 1 : 200, 1 : 300, 1 : 400 and 1 : 500 were studied. Both the background noise and target signal reduced as the dilution ratio increased, and the declining tendency gradually slowed to a plateau since 1 : 200. As shown in Fig. 2d, all three assays achieved the best S/N ratio at 1 : 200. The S/N ratio at 1 : 100 was lower than that at 1 : 200, because the dosage of SAPE at 1 : 100 was greater than the actual requirement, causing extra background noise. The differences between the background noise and target signal tended to fade away with the further increase in the dilution ratios, which can be attributed to the fact that the amount of SAPE is less than that actually needed. In the end, 1 : 200 was applied for the final assays.

Admittedly, there were S/N fluctuations during the optimization process. Especially, S/N values in Fig. 2c were found to be lower overall than those in the other three figures due to the higher background noise occurring in the optimization experiment of capture probes. The reasons for the batch-to-batch differences could be attributed to the following points. On the one hand, the effect of wash steps may be the biggest factor. Although we had tried our best to wash thoroughly in every experiment, there was still no guarantee that the effect of wash steps in each experiment would be the same. Besides, different batches of synthesized microspheres themselves may bring different background noise.

### Specificity test

**Fig. 3:**
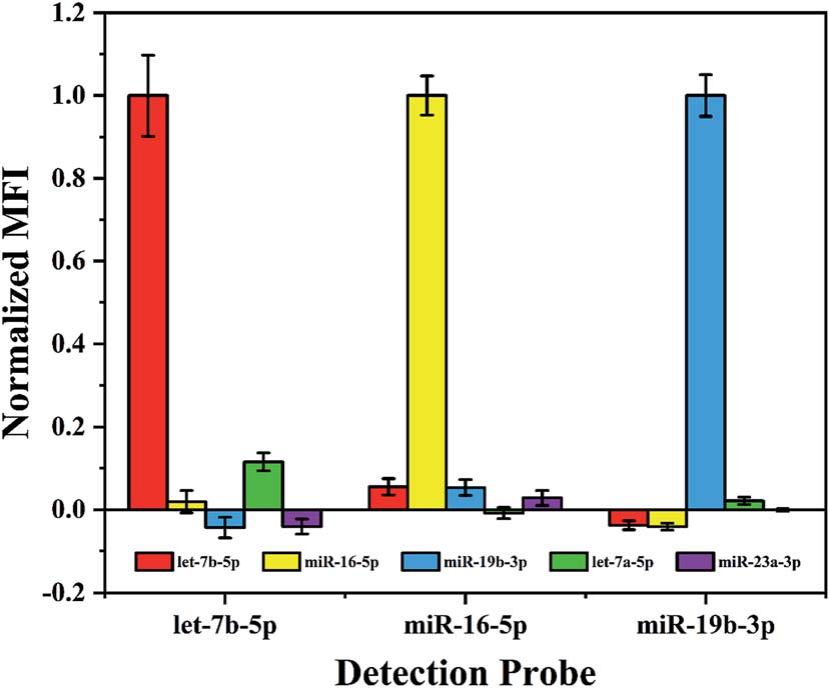
Specificity test. Normalized MFI denotes the further normalized value of 5 pM target or interfering miRNAs captured respectively by the three detection probes against the control signal. Experimental conditions: RNase dilution 1 : 10, chimeric probe concentration 0.01 μM, capture probe coupling concentration 100 pmol/10^6^ microspheres, SAPE dilution 1 : 200 and microspheres density 10^4^ microspheres per well.

In a multiplex assay for the detection of three miRNA targets mentioned above, cross-hybridization may occur among these different miRNAs and detection probes in the detection system. Therefore, before performing such assay the specificity of the proposed method needs to be evaluated. In order to simulate the interference of other miRNAs in the real sample, in addition to the existing three miRNAs, let-7a-5p with a single base mismatch and a random sequence miR-23a-3p (see Table 1) were introduced to the hybridization samples. As shown in Fig. 3, the normalized MFI from the non-target miRNA is about 0.1, it suggests that this multiplex assay performs excellent specificity even to recognize single base mismatch.

### Multiplex assay of target miRNAs

**Fig. 4:**
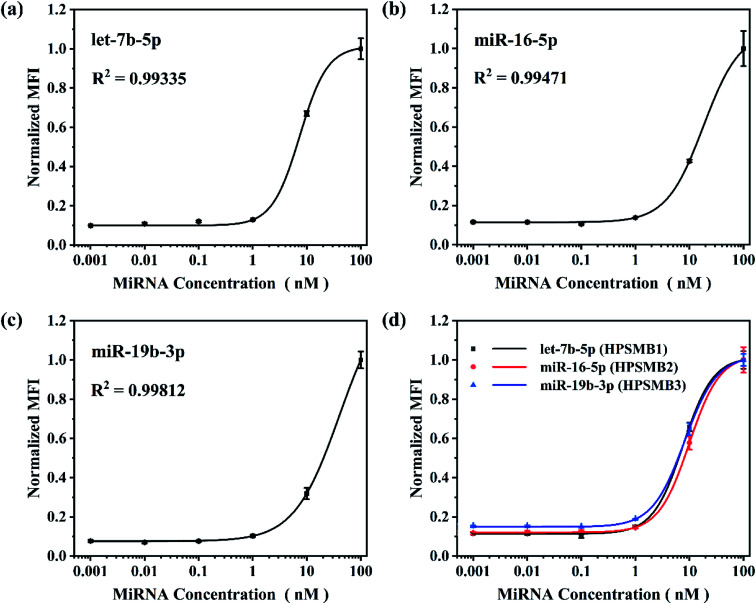
Single assay calibration plots and triplex multiplex assay standard curves. Normalized MFI equals to normalized value of target signal. Experimental conditions: (a–c) RNase dilution 1 : 10, chimeric probe concentration 0.01 μM, capture probe coupling concentration 100 pmol/10^6^ microspheres, SAPE dilution 1 : 200 and microspheres density 10^4^ microspheres per well. (d) RNase dilution 3 : 10, each chimeric probe concentration 0.01 μM, each capture probe coupling concentration 100 pmol/10^6^ microspheres, SAPE dilution 3 : 200 and each microspheres density 10^4^ microspheres per well.

Based on the optimized reaction parameters, single assay for the three plasma biomarkers of gastric cancer, named let-7b-5p, miR-16-5p and miR-19b-3p was performed. As shown in Fig. 4a–c, *R*^2^ values were achieved between 0.993 and 0.998, showing good correlations of normalized MFI and target miRNA concentrations from 0.001 to 100 nM in all three miRNA assays. After that, triplex hybridization assay was performed by using mixed HPSMBs with three different fluorescent barcodes. As shown in Fig. 4d, all the three miRNAs present good correlations of normalized MFI with miRNA concentrations like the results in the single-plex assay. The LODs of let-7b-5p, miR-16-5p, miR-19b-3p in a multiplex assay are 0.43, 0.76 and 0.60 nM, respectively. In this multiplex assay, three different encoded microspheres were utilized to address each kind of miRNA individually, and the normalized MFI of each kind of miRNA showed a similar trend. It indicates that encoded microspheres as the qualitative indicator do not cause additional interference to the quantitative results. Besides, the LODs of the three miRNAs in the multiplex assay are similar to those in the single assay, which indirectly proves the good specificity of this detection assay. More importantly, our platform could be extended to detect more other target miRNAs simultaneously based on more different encoded microspheres, whose fluorescent barcodes could be enriched by changing the amounts and colors of AIEgens inside PSMA microspheres, changing the size of microspheres, thus making it more powerful for multiplex detection in further clinical diagnosis.

## Conclusion

A novel suspension array platform based on AIEgens-encoded microspheres for miRNA multiplex detection was firstly constructed, wherein AIEgens-encoded microspheres were prepared by incorporating HPS into PSMA microspheres through using SPG membrane emulsification method. The as-prepared HPSMBs present high uniformity, bright fluorescent intensity and good distribution of HPS inside the microspheres resulting from the unique advantages of SPG membrane emulsification technique and excellent optical properties of HPS. Triplex detection assay based on suspension array platform with HPSMBs was performed for detecting let-7b-5p, miR-16-5p and miR-19b-3p as gastric cancer plasma biomarkers, demonstrating the feasibility of our miRNA multiplex assay platform. The results show that this multiplex assay has good specificity able to recognize single base mismatch, and high sensitivity with LODs ranging from 0.43 to 0.76 nM for the three miRNAs. Moreover, our method could be extended to the simultaneous detection of more other target miRNAs by designing specific capture probes and chimeric probes based on the developed method. With flexibility, strong multiplexed ability and good detection performance, it could hold good potential in further clinical diagnosis.

## Authors' contributions

Zou was responsible for the design of detection probes, experiments performing, data processing, and manuscript drafting. Wu mainly helped to design the experiment, prepared AIEgens microspheres and modified the manuscript. Zhang helped to discuss problems in the experiments and modify the manuscript. Qiang Ma collected clinical specimens and tested this method. Other authors helped to discuss the experiments and review the manuscript. All authors read and approved the final manuscript.

## Conflicts of interest

There are no conflicts to declare.

## Supplementary Material

RA-009-C9RA07680H-s001
