# Supplementary material for: Multiplex detection of miRNAs based on aggregation-induced emission luminogen encoded microspheres
Source: RSC Adv. 2019 Dec 4;9(68):39976–85. doi: 10.1039/c9ra07680h (PMC9076168; doi:10.1039/c9ra07680h)
Supplement: RA-009-C9RA07680H-s001 [file RA-009-C9RA07680H-s001.pdf]

Electronic Supplementary Material (ESI) for RSC Advances.
This journal is © The Royal Society of Chemistry 2019

Supporting Information

Multiplex Detection of miRNAs based on Aggregation-Induced Emission Luminogens Encoded Microspheres

Dan Zou[ab#], Weijie Wu[c#], Jingpu Zhang[d], Qiang Ma[e], Sisi Fan[a], Jin Cheng[a], Dan Li[c], Jiaqi Niu[ab], Xiaoqing Qian[ab], Wanwan Li*[c], Daxiang Cui*[ab]

# Zou and Wu contributed equally to this work.

* Corresponding author

a. Institute of Nano Biomedicine and Engineering, Shanghai Engineering Research Center for Intelligent Instrument for Diagnosis and Therapy, Department of Instrument Science & Engineering, School of Electronic Information and Electrical Engineering, Shanghai Jiao Tong University, 800 Dongchuan RD, Shanghai 200240, China.

E-mail address: dxcui@sjtu.edu.cn

b. National Center for Translational Medicine, Collaborative Innovational Center for System Biology, Shanghai Jiao Tong University, Shanghai 200240, P. R. China.

c. State Key Lab of Metal Matrix Composites, School of Materials Science and Engineering, Shanghai Jiao Tong University, 800 Dongchuan Road, Shanghai 200240, P. R. China.

E-mail address: wwli@sjtu.edu.cn

d. Scientific Research Center, Shanghai Public Health Clinical Center, Fudan University, 2901 Caolang Road, Shanghai, 201508, P.R. China.

e. Tumor Department of Xintai People Hospital, 1329 Xinfu Road, Xintai City, Shandong Province, China

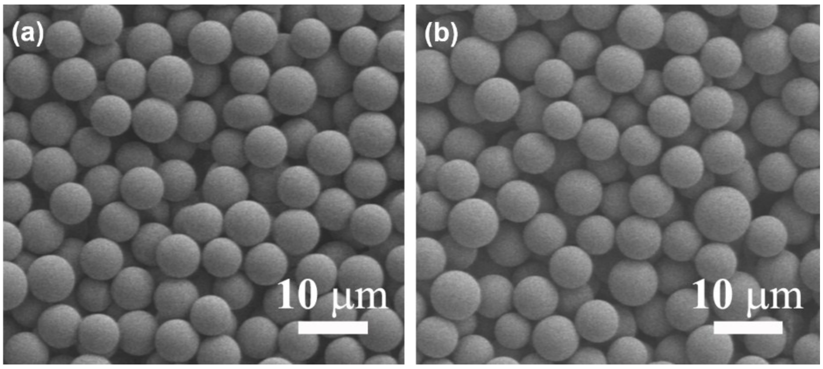

Figure Ⅰ. SEM images of HPSMBs 1 and HPSMBs 3, respectively.

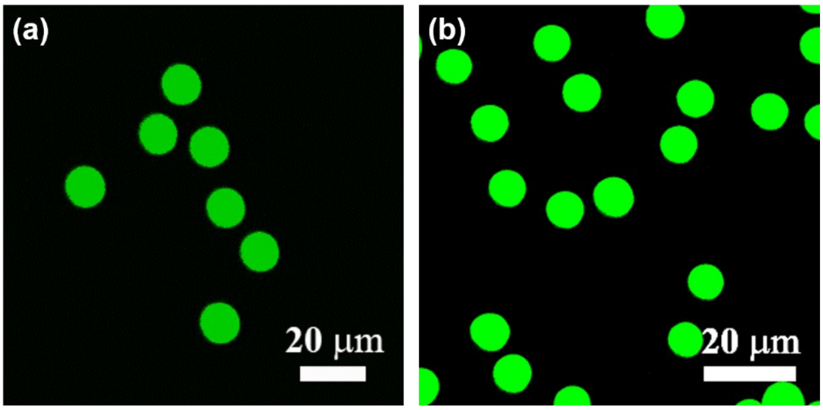

Figure Ⅱ. CLSM images of HPSMBs 1 and HPSMBs 3, respectively.
